# Characterization and Modeling of a Pt-In_2_O_3_ Resistive Sensor for Hydrogen Detection at Room Temperature

**DOI:** 10.3390/s22197306

**Published:** 2022-09-26

**Authors:** Meile Wu, Zebin Wang, Zhanyu Wu, Peng Zhang, Shixin Hu, Xiaoshi Jin, Meng Li, Jong-Ho Lee

**Affiliations:** 1 School of Information Science and Engineering, Shenyang University of Technology, Shenyang 110870, China; 2 School of Electrical Engineering, Nanjing Vocational University of Industry Technology, Nanjing 210023, China; 3 Shenyang Institute of Automation, Chinese Academy of Sciences, Shenyang 110016, China; 4 Department of Electrical and Computer Engineering and Inter-University Semiconductor Research Center (ISRC), Seoul National University, Seoul 08826, Korea

**Keywords:** hydrogen sensing, In_2_O_3_, Pt doping, room temperature, modeling, inkjet printing

## Abstract

Sensitive H_2_ sensors at low concentrations and room temperature are desired for the early warning and control of hydrogen leakage. In this paper, a resistive sensor based on Pt-doped In_2_O_3_ nanoparticles was fabricated using inkjet printing process. The H_2_ sensing performance of the sensor was evaluated at low concentrations below 1% at room temperature. It exhibited a relative high response of 42.34% to 0.6% H_2_. As the relative humidity of 0.5% H_2_ decreased from 34% to 23%, the response decreased slightly from 34% to 23%. The sensing principle and the humidity effect were discussed. A dynamic current sensing model for dry H_2_ detection was proposed based on Wolkenstein theory and experimentally verified to be able to predict the sensing behavior of the sensor. The H_2_ concentration can be calculated within a short measurement time using the model without waiting for the saturation of the response, which significantly reduces the sensing and recovery time of the sensor. The sensor is expected to be a promising candidate for room-temperature H_2_ detection, and the proposed model could be very helpful in promoting the application of the sensor for real-time H_2_ leakage monitoring.

## 1. Introduction

Hydrogen fuel is regarded as a vital green energy to support the operation of human society in the future due to its non-polluting combustion and high energy conversion rate [1,2]. However, the flammable and explosive properties of H_2_ and the broad explosive limit concentration range of 4–75% are serious safety concerns in the development of hydrogen energy [3]. Since hydrogen gas (H_2_) is colorless and odorless, it is challenging to detect it in time, especially at low concentrations and room temperature [4,5]. Therefore, it is of great significance to develop sensors that can detect H_2_ sensitively and reliably at room temperature for the early warning and control of H_2_ leakage.

Resistive H_2_ sensors based on metal oxide semiconductors (MOS) have been widely reported because of their simple fabrication, low cost, and good selectivity. At present, many MOS materials, such as WO_3_ [6], In_2_O_3_ [7], TiO_2_ [8], ZnO [9], and SnO_2_ [10], have been used in the detection of H_2_. Among them, In_2_O_3_, an n-type semiconductor with a wide-bandgap (~3.6 eV), shows great potential due to its low cost, high electrical conductivity, high stability, and excellent H_2_ sensitivity [11]. Various forms of low-dimensional In_2_O_3_ have been studied to meet the requirements of room temperature and fast and stable H_2_ detection [12,13]. The nanostructure enables the MOS materials to have a high surface/volume ratio and an enhanced gas adsorption capacity [14,15,16]. Additionally, doping noble metals, such as Au, Ag, Pd, Pt, etc., was used as another effective method to improve the sensing performance of materials, since the catalysis of noble metals can promote the sensing reactions [17,18,19,20,21].

In this paper, In_2_O_3_ nanoparticles with a diameter of about 100 nm doped by 10-weight-concentration (wt.%) Pt were used as the sensitive material for the detection of H_2_ at room temperature. Platinum was adopted as the dopant because it has a lower hydrogen-solubility compared with other commonly used inert metals, which is more beneficial to improving the stability of the sensor [20,22]. A precursor solution containing In_2_O_3_ nanopowders and H_2_PtCl_6_ was deposited by inkjet printing technology on the integrated electrodes and was heated to form the sensing layer. The adopted inkjet printing technology makes the fabrication process of the sensor simple and convenient with good repeatability and mass production capability. Besides, the heating temperature is controlled at 300 °C, which will not affect other semiconductor devices when the sensor is integrated with other functional circuits on the same silicon wafer for forming a sensing system. Sensing behaviors of the proposed sensor for both dry and humid H_2_ were characterized in this work, which presented a reasonable H_2_ sensing properties at room temperature. Moreover, the resistive sensor with nano Pt-In_2_O_3_ was also modeled and analyzed in order to deeply understand the H_2_ sensing principle at room temperature and help to predict the sensing results of the sensor. The method mainly relies on analytical descriptions of the adsorption and desorption behavior (Wolkenstein and Langmuir theories) and energy band theory of semiconductors. A model of transient sensing characteristics of the proposed sensor was built to predict the dynamic change in the current response to H_2_ of the sensor and validated experimentally. It also contributed to explain the humidity effect on H_2_ sensitivity of the sensor. In this work, the Pt-In_2_O_3_ sensor could be a promising candidate for room-temperature H_2_ detection, and the proposed model could be very helpful in promoting the application of the sensor for real-time H_2_ leakage monitoring and the establishment of complex gas sensing systems by integrating with circuits.

## 2. Materials and Methods

The resistive sensor platform was fabricated on a silicon wafer with an O/N/O passivation layer consisting of SiO_2_ (10 nm)/Si_3_N_4_ (20 nm)/SiO_2_ (10 nm). The Cr (30 nm)/Au (50 nm) multilayer was deposited and patterned to serve as the interdigitated electrodes. The width of the electrodes and the space between them are 2 µm and 1.8 µm, respectively. Then, an SU-8 passivation layer formed by spin coating was patterned on top of the wafer by a lithography process to expose only the interdigitated-electrode area and the contact pads of the of resistor platform, which can protect other devices on the wafer.

The Pt-In_2_O_3_ sensitive material was formed by an inkjet printing process. Firstly, In_2_O_3_ nanopowders (about 100 nm in diameter) were dissolved in ethanol (99%) and stirred thoroughly. Next, an 8 wt.% H_2_PtCl_6_ (in H_2_O) solution was further diluted by deionized water to the desired concentration and mixed with the In_2_O_3_ solution together to serve as the precursor ink. The as-prepared ink was printed on the interdigitated-electrode area of the platform using Omni Jet 100 inkjet printer, followed by a 2-h annealing process at 300 °C in air to fully evaporate the solvent and decompose H_2_PtCl_6_ into Pt [23]. The wt.% of Pt in the sensing layer was set to be 10 wt.% to focus principally on the analysis of hydrogen adsorption and effects of moisture on the sensing properties. The resistance of the obtained sensor is about 7.3 × 10^5^ kΩ. All chemicals used in this paper were purchased from Sigma-Aldrich without further purification.

**Figure 1:**
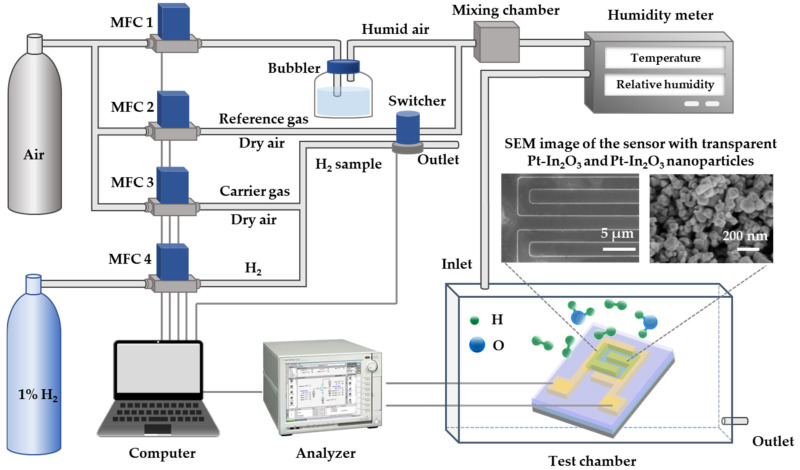
The experimental setup for both dry and humid H_2_ detection at room temperature.

The experimental setup is shown in Figure 1. In this paper, the prepared sensor was characterized for both dry and humid H_2_ detection. During the test, the sensor was placed in the test chamber, and the total flow rate of the gas flowing into the chamber was always maintained at 400 sccm. In the tests of dry H_2_ detection, MFC 1 in Figure 1 was off. In the initial stage, dry air served as reference gas (400 sccm) and was controlled by MFC 2 to blow into the chamber, and the sensor was in a standby state. When starting to sense H_2_, the reference gas was switched to the sample gas with a fixed concentration of H_2_ (400 sccm) injected into the test chamber by using the switcher in Figure 1. At the same time, the reference gas was converted to the outlet and discharged into the atmosphere. Hydrogen gas samples were prepared by diluting 1% H_2_ with a dry carrier gas (air) to desired concentrations using MFC 3 and MFC 4.

In the tests of humid H_2_ detection, MFC 1 was turned on to let another stream of dry air flow into the bubbler and then mixed with the reference dry air or the dry H_2_ samples to bring water vapor into the test chamber. The total flow rate of the gas injected into the chamber was still fixed at 400 sccm, and the relative humidity (RH) was monitored by the humidity meter in Figure 1 and tuned by MFC 1 to a desired level. Figure 1 also presented SEM images of the sensor with a transparent Pt-In_2_O_3_ layer and Pt-In_2_O_3_ layer nanoparticles. A clear magnification of the SEM image of Pt-In_2_O_3_ particles in Figure 1 and EDS analysis are provided in Figure S1 of the Supplementary Materials which confirms the formation of Pt-In_2_O_3_. During all sensing tests, the voltage between the two electrodes of the sensor was fixed to measure the changes in current that corresponded to the variation in the H_2_ concentration. All sensing characteristics of all sensors were tested at 25 °C (room temperature). Electrical measurements were carried out by using an Agilent B1500A analyzer.

## 3. Results

### 3.1. Experimental Results

**Figure 2:**
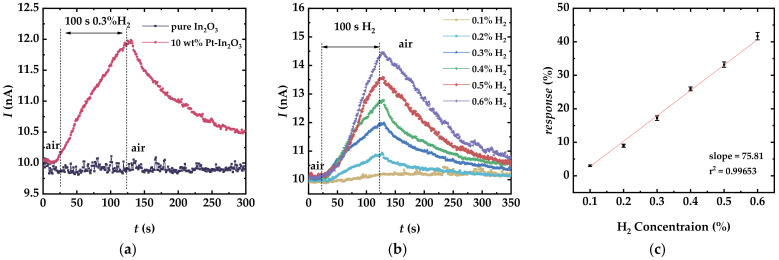
Results from dry H_2_ sensing tests performed with the Pt-In_2_O_3_ resistive sensor exposed to H_2_. (**a**) Comparison between the resistive sensors with pure In_2_O_3_ and 10 wt.% Pt-In_2_O_3_ for the detection of 0.3% H_2_. (**b**) Dynamic response of the sensor towards different concentrations of H_2_ in a range of 0.1–0.6% at room temperature. A determined concentration of dry H_2_ gas was introduced in the period of 20–120 s. (**c**) Sensor response as a function of H_2_ concentration. Each error bar corresponds to one standard deviation considering the uncertainty of three response values.

Firstly, a comparison between the resistive sensors with pure In_2_O_3_ and 10 wt.% Pt-In_2_O_3_ for the detection of 0.3% H_2_ was conducted as shown in Figure 2a, which indicates that Pt was well-formed on the surface of In_2_O_3_ and improved the H_2_ sensitivity of the sensor. Then, dry H_2_ sensing performance of the Pt-In_2_O_3_ resistive sensor was measured. The results are shown in Figure 2b,c. In the initial stage of tests, 400 sccm of dry air was blown towards the sensor. From 20 s in Figure 2b, the air was replaced by a dry H_2_ sample with a certain concentration and maintained for 100 s for the sensor to respond. After that (at 120 s in Figure 2b), the gas flowing into the chamber was changed back to the reference gas, and the sensor entered the recovery period. The above experimental procedure was repeated six times, and the H_2_ concentration used each time was set from 0.1% to 0.6%, respectively, with a step of 0.1%. The responses, calculated by using Equation (1), are 3.04%, 9.25%, 17.60%, 26.07%, 33.69%, and 42.34% for 0.1–0.6% of H_2_, respectively, based on the data in Figure 2a.

(1) $$Response=\left[\left(I_{H}-I_{\mathrm{air}}\right)/I_{\mathrm{air}}\right]\times 100\%$$

where *I*_H_ and *I*_air_ denote the current of the sensor corresponding to H_2_ samples and reference gas, respectively. The response and recovery times of the sensor were plotted as functions of H_2_ concentration in Supplementary Figure S2. According to the results, the response of the sensor was very limited for the 0.1% H_2_. With the increasing H_2_ concentration, the rising speed of the sensor current increased significantly. Figure 2c plots the response of the Pt-In_2_O_3_ sensor as a function of H_2_ concentration, showing a near-linear relationship. Each error bar corresponds to one standard deviation, considering the uncertainty of three response values.

The detection limit (DL) was also calculated here, as it is a crucial indicator reflecting the sensing performance for practical application [24]. First, the standard deviation in the baseline of the response curve (rms_noise_) was calculated from 20 data points, which is 0.003828, representing the level of the sensor noise. The slop of the linear fitting of gas response versus gas concentration at RT is 75.81454 according to Figure 2c. Therefore, the DL = 3rms_noise_/slop = 1.51 ppm according to literature [24,25]. Additionally, a comparison between the proposed sensor of this work and recent H_2_ sensors based on various sensitive materials was conducted as shown in Table 1 [6,26,27,28,29,30,31,32,33,34].

**Figure 3:**
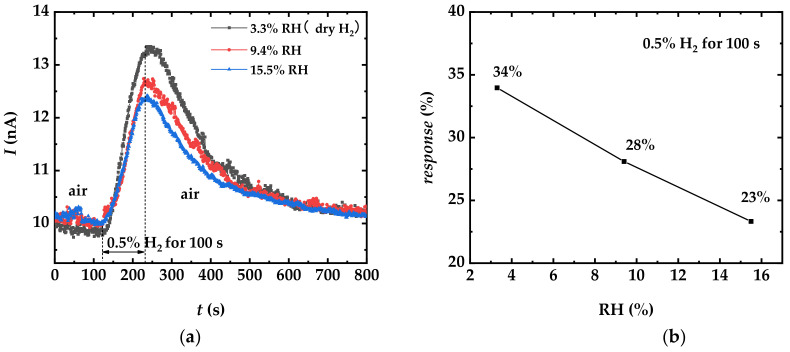
Results from humid H_2_ sensing tests performed with the Pt-In_2_O_3_ resistive sensor exposed to H_2_. (**a**) Dynamic response of the sensor towards 0.5% H_2_ at 3.3% RH (corresponding to dry H_2_), 9.4% RH, and 15.5% RH, respectively. The response decreases slightly with the increasing RH. (**b**) Sensor response as a function of RH for 0.5% H_2_.

As the aim of this work is the detection of H_2_ at room temperature, it is necessary to consider the effect of humidity on the sensor performance. Therefore, humid H_2_ sensing tests were also conducted and the measurement results are shown in Figure 3. The experimental setup and gas controls can be found in Section 2.

Figure 3a shows the dynamic response of the sensor towards 0.5% H_2_ at 3.3% RH (corresponding to dry H_2_), 9.4% RH, and 15.5% RH, respectively. The humid H_2_ samples were maintained for 100s (from 120 s to 220 s in Figure 3a), and the change in current at 220 s was slightly reduced by the increasing RH. Figure 3b shows the variation of the response with RH for 0.5% H_2_. As the RH increased from 3.3% to 15.5%, the response decreases from 34% to 23%. The results illustrate that water vapor deteriorates the hydrogen sensitivity of the device. The rationale behind this was explored in-depth in the next section.

### 3.2. Modeling and Computation

#### 3.2.1. Current Model

In this section, by analyzing the sensing principle of the prepared sensor, a numerical sensing model was proposed based on adsorption kinetics and energy band theory and validated by experimental data. Finally, the proposed modeling approach was used to explain the mechanism of the humidity effect on the H_2_ sensing performance of the inkjet-printed Pt-In_2_O_3_ sensor.

**Figure 4:**
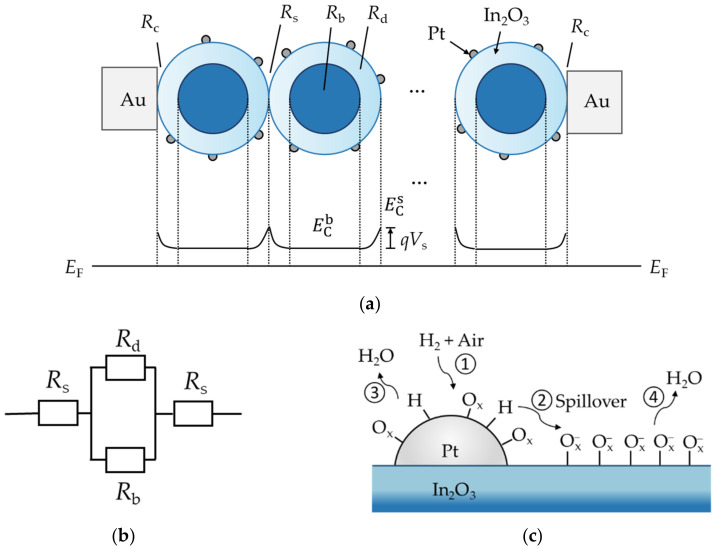
Schematic diagram of the Pt-In_2_O_3_ resistive sensor. (**a**) Simplified structure and energy band diagram, where *R*_c_, *R*_s_, *R*_b_, and *R*_d_ are the contact resistance between the metal electrode and the sensitive material, the surface resistance at the interface between In_2_O_3_ particles, the bulk resistance inside the particle, and the resistance of the depletion region at the periphery of the particle, respectively; *E*_F_ is the Fermi energy level, and $E_{C}^{b}$ and $E_{C}^{s}$ are the conduction bands of the bulk and surface of the In_2_O_3_ particle, respectively. When the sensor is placed in an air atmosphere, O_2_ naturally adsorbs on the surface of the In_2_O_3_, forming an upward bending of the surface energy band denoted as *qV*_s_ (*V*_s_ < 0); (**b**) Composition of the resistance of an In_2_O_3_ particle after simplification. (**c**) Schematic diagram illustrating the H_2_ sensing mechanism of Pt-In_2_O_3_. Here, ①, ②, ③, and ④ represent the dissociative adsorption process of hydrogen at Pt, the spillover process, the direct reaction with adsorbed oxygen species on Pt to produce water vapor, and the reaction with adsorbed oxygen species on the surface of In_2_O_3_ to produce water vapor and release electrons, respectively; O_x_ and $O_{x}^{-}$ represent the possible oxygen species adsorbed on Pt and In_2_O_3_.

For the sake of illustration, the Pt-In_2_O_3_ resistive sensor was simplified to the structure shown in Figure 4a, i.e., two metal electrodes are connected by spherical In_2_O_3_ nanoparticles embedded with Pt on the surface. In the figure, *R*_c_ is the contact resistance between the electrode and the sensitive material, *R*_s_ is the surface resistance at the interface between In_2_O_3_ particles, *R*_b_ and *R*_d_ are the resistance of the bulk and surface depletion region of an In_2_O_3_ particle, respectively; *E*_F_ is the Fermi energy level, $E_{C}^{b}$ and $E_{C}^{s}$ are the conduction bands of the bulk and surface of the In_2_O_3_ particle, respectively. At room temperature, the catalytic effect of Pt causes the adsorption of oxygen on the surface of In_2_O_3_ particles in the form of $O_{x}^{-}$, as shown in Figure 4c (such as O^−^ and $O_{2}^{-}$ at low temperature) [35,36]. During this process, electrons in In_2_O_3_ near the surface transfer to the adsorbed oxygen species to form a space charge region (depletion of electrons) according to a possible reaction shown as Equation (2) [37], which induces upward bending of the energy bands at the surface denoted as *qV*_s_ (*V*_s_ < 0).

(2) $$O_{x\left(\mathrm{ads}\right)}+e^{-}\to O_{x\left(\mathrm{ads}\right)}^{-}$$

The average diameter of the In_2_O_3_ particles used in this paper is 100 nm, which is larger than the Debye length, so it can be considered that the particles are in a partially depleted state. That is, there is a low-resistance electron accumulation region in the body of the particle, and only the surface is depleted by the chemisorption of oxygen. The thickness of the depletion region changes accordingly with the type and concentration of the gas molecules adsorbed on the surface and affect the overall resistance of the particle. Based on these analyses, the resistance of an In_2_O_3_ particle can be simplified to the composition shown in Figure 4b. In the middle part of the particle, the resistances of the bulk and the surface depletion region are in parallel, and they connect with a surface resistance in series on each side of the particle, i.e.,

(3) $$R=2R_{s}+\frac{R_{d}\cdot R_{b}}{R_{d}+R_{b}}$$

As In_2_O_3_ is an n-type semiconductor, the total resistance of a particle can be written as Equation (4) according to literature [38].

(4) $$R_{p}\propto R_{b}\cdot e^{-\frac{qV_{s}}{kT}}$$

Note that the contribution of *R*_c_ to overall resistance of the sensor and the change of it by gas adsorption can be ignored [38,39]. Thus, the entire sensor can be approximately considered to be composed of numerous *R*_p_s in series and parallel, i.e.,

(5) $$R\approx \gamma \cdot R_{b}\cdot e^{-\frac{qV_{s}}{kT}}$$

where γ is the coefficient related to the morphology of the sensitive material, the contact area between the particles, and the geometry chosen for simulating the particle (such as sphere, cylinder, and cube, etc.). Thus, the current can be written as

(6) $$I=\frac{U}{R}=\frac{U}{\gamma \cdot R_{b}}\cdot e^{\frac{qV_{s}}{kT}}=\beta \cdot e^{\frac{qV_{s}}{kT}}$$

where, $\beta =U/(\gamma \cdot R_{b})$, and *U* is the applied external DC voltage.

Figure 4c shows the schematic diagram of the H_2_ sensing mechanism of Pt-In_2_O_3_. When the sensor is exposed to H_2_, the H_2_ molecules can be dissociated into H atoms by the catalytic effect of Pt even at room temperature according to a possible reaction shown as Equation (7) [22], which corresponds to process ① in the figure.

(7) $$H_{2}\overset{\mathrm{Pt}}{\to }2H_{\left(\mathrm{ads}\right)}$$

The difference in H concentration between the surfaces of Pt and In_2_O_3_ leads to the diffusion of H atoms from Pt to In_2_O_3_, which is so-called spillover corresponding to process ②. Process ③ includes two reactions. On one hand, before diffusing to In_2_O_3_, hydrogen atoms can react with the adsorbed oxygen species which have already existed on Pt in the standby stage of the sensor, thereby generating water vapor leaving the surface of the sensitive material. On the other hand, as the H_2_ gas sample is a mixture of 1 % H_2_ and air, it contains both H_2_ and O_2_, which can also react directly under the catalysis of Pt to produce water molecules when both gases meet with each other on the surface of Pt. When H atoms diffuse to In_2_O_3_, they react with the negatively charged oxygen species to generate water and release free electrons to In_2_O_3_, which is the reduction reaction that plays a major role in H_2_ detection and denoted by ④ in Figure 4c. As a result, H_2_ reduces the thickness of the depletion layer on the surface of In_2_O_3_, thereby reducing the |*V*s| and increasing the current.

#### 3.2.2. Hydrogen Dynamic Sensing Model under Dry Conditions

**Figure 5:**
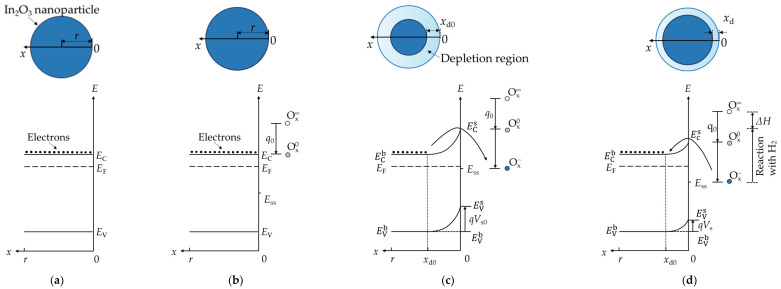
Schematic diagram of the energy band distributions and the electron distributions of In_2_O_3_ particles based on the Wolkenstein adsorption theory (Note that Pt was not indicated in this figure). (**a**) An ideal pure In_2_O_3_ particle. (**b**) The particle under weak chemisorption of oxygen. (**c**) The particle with strong chemisorbed oxygen ($O_{x}^{-}$) after thermal equilibrium and electron depletion near the surface. (**d**) The particle undergoing a reduction reaction after the presence of hydrogen. *E*_C_, *E*_F_, and *E*_V_ represent the conduction band, Fermi energy level, and valence band, respectively. The superscripts s and b represent the parameters of the surface and the core of an In_2_O_3_ particle, respectively. The binding energy of weak chemisorption is denoted as *q*_0_. $O_{x}^{\infty }$, $O_{x}^{0}$, and $O_{x}^{-}$ are the oxygen species before adsorption, weakly adsorbed, and strongly adsorbed, respectively. *ΔH* represents the difference between the adsorption energy of oxygen species and the chemical energy of the reaction between the sensitive material and H_2_, *r* is the average radius of indium oxide particles, and *x*_d0_ and *x*_d_ are the thickness of the depletion layer at oxygen adsorption equilibrium and after the reaction with hydrogen, respectively.

There are two commonly used theories for the analytical descriptions of the adsorption and desorption behavior, which are Langmuir and Wolkenstein theories, respectively. The Langmuir model assumes that the bonding energy between the adsorbent and the adsorbed material is a fixed constant. However, both the oxygen adsorption and the H_2_ detection involved in this paper are related to chemical reactions, and the processes include electron transfer between the adsorbent and the sensitive material. In this case, the binding energy or adsorption heat will vary with the coverage of the chemisorbed material, so that the Langmuir model cannot meet the requirements for establishing the dynamic adsorption model here. The Wolkenstein model, by contrast, takes into account both the electron interactions and their effects on the adsorption properties of the semiconductors. Therefore, the Wolkenstein model was adopted here. Figure 5 shows the energy band distributions and the corresponding internal and surface electron distributions of In_2_O_3_ particles based on the Wolfenstein theory for four cases of pure surface (a), weak oxygen adsorption, (b) strong oxygen adsorption at equilibrium state (c), and hydrogen reduction (d). It is worth noting that Pt is not marked in the figure. The doped Pt and In_2_O_3_ were studied as a whole in this section.

The core of Wolkenstein’s model is to divide chemisorption of gases into two steps: weak chemisorption and strong chemisorption. In the ideal situation, the energy bands of the In_2_O_3_ particle with an empty surface are assumed to be flat, as shown in Figure 5a. By regarding the In_2_O_3_ particles as spheres, a coordinate axis *x* was established along the surface to the center of the sphere. Next, the energy band distribution was obtained at the bottom of Figure 5a, where *r* is the average radius of the particles. The energy bands of the particles are consistent from the inside to the outside, and the electrons are evenly distributed throughout the sphere. *E*_C_, *E*_F_, and *E*_V_ represent the conduction band, Fermi energy level, and valence band, respectively.

When the sensor is exposed to a dry air, oxygen will be dissociated into O or adsorbed molecularly in the presence of Pt and diffuse to the surface of In_2_O_3_ at low temperature [35,36,40]. As shown in Figure 4b, when $O_{x}^{\infty }$ reaches the indium oxide surface, a weak chemisorption without electron transfer is formed firstly, so that the energy bands are still flat and the concentration of electrons throughout the particle is still the same. During this process, the state of O species changes from $O_{x}^{\infty }$ to $O_{x}^{0}$, and the adsorption energy is *q*_0_. At the same time, an additional surface state (*E*_ss_) is also introduced in the bandgap of In_2_O_3_.

Since the Fermi energy level is higher than *E*_ss_, the electrons in the conduction band bottom of In_2_O_3_ oxide are taken away by $O_{x}^{0}$ and the weakly adsorbed $O_{x}^{0}$ is further strongly chemisorbed to form $O_{x}^{-}$ ions under the room-temperature test condition of this paper [35]. Thus, at equilibrium, the energy bands bend upward, as shown in Figure 5c. At this point, the conduction band and valence band at the surface ($E_{C}^{s}$ and $E_{V}^{s}$) are no longer the same as the ones in the bulk ($E_{C}^{b}$ and $E_{V}^{b}$). The band bending is recorded as *qV*_s0_ (*V*_s0_ < 0), which leads a depletion of electrons near the surface of In_2_O_3_ with a thickness of *x*_d0_. The total adsorption energy of O dissociated by Pt on the In_2_O_3_ is *q*_0_ + $E_{C}^{s}\;$ − *E*_ss_.

When the sensor starts to detect H_2_, H_2_ molecules will dissociate into H atoms under the catalysis of Pt, come to the surface of In_2_O_3_ through the spillover process, and react with $O_{x}^{-}$ species, thereby returning electrons to the conduction band of In_2_O_3_. Therefore, the upward bending degree of the energy bands is reduced from *qV*_s0_ to *qV*_s_, and the thickness of the depletion region is reduced from *x*_d0_ to *x*_d_, as shown in Figure 5d. If ΔH is denoted as the difference between the oxygen adsorption energy and the hydrogen reaction energy, the hydrogen reaction energy can be expressed as *q*_0_ +$\;E_{C}^{s}\;$ − *E*_ss_ − ΔH.

It is assumed that the O^−^ species on the surface of In_2_O_3_ plays the predominant role during the sensing process [35] and the concentration is *N^−^*. In the standby state of the sensor, the adsorption of O^−^ is in equilibrium. When H_2_ appears, *N^−^* is changed by the desorption of O^−^ and the reduction reaction with hydrogen with time, which can be written as

(8) $$\frac{dN^{-}}{dt}=O\left(N^{-}\right)+H\left(N^{-}\right)$$

Assuming that the reduction reaction with hydrogen is the dominant factor effecting the concentration of O^−^ on the surface of In_2_O_3_, then Equation (9) can be obtained as below according to Wolkenstein theory [41],

(9) $$\frac{dN^{-}}{dt}=-\alpha_{1}P_{H}N^{-}e^{-\frac{q_{0}-\cdot H+\left(E_{C}^{b}-E_{ss}\right)}{kT}}$$

where α_1_ is the rate constant of the reaction, *k* is the Boltzmann constant, *T* is the thermodynamic temperature, and *P*_H_ is the partial pressure of H_2_. As it is known that $E_{C}^{b}$ =$\;E_{C}^{s}$ + *qV*_s_ ($0>V_{s}>V_{s0}$), and *P*_H_ ∝ *C*_H_, where *C*_H_ is the concentration of H_2_, then Equation (9) can be rewritten as

(10) $$\frac{dN^{-}}{dt}=-\alpha_{1}^{\prime }C_{H}N^{-}e^{-\frac{q_{0}-\cdot H}{kT}}\cdot e^{-\frac{E_{C}^{s}-E_{ss}}{kT}}\cdot e^{-\frac{qV_{s}}{kT}}$$

where $\alpha_{1}^{\prime }$ is the coefficient related to *P*_H_ and *C*_H_. Then, Equation (10) is rewritten as

(11) $$\frac{dN^{-}}{dt}=m\cdot C_{H}\cdot N^{-}\cdot e^{-\frac{qV_{s}}{kT}}$$

with

(12) $$m=-\alpha_{1}^{\prime }e^{-\frac{q_{0}-∆H}{kT}}\cdot e^{-\frac{E_{C}^{s}-E_{ss}}{kT}}$$

According to the full depletion approximation of the space charge region in semiconductor physics, i.e., there are no mobile carriers in the space charge region, the thickness of the depletion layer and the corresponding *N^−^* at a certain moment are obtained as follows

(13) $$x_{d}={\left(\frac{-2\varepsilon }{qn_{b}}\cdot V_{s}\right)}^{\frac{1}{2}}$$

(14) $$N^{-}=n_{b}\cdot x_{d}={\left(-\frac{2\varepsilon n_{b}}{q}\cdot V_{s}\right)}^{\frac{1}{2}}$$

where *n*_b_ is the concentration of electrons inside the In_2_O_3_ nanoparticle, and *ε* is the dielectric constant of the sensitive material. Substituting Equation (14) into 11 yields

(15) $$\frac{dN^{-}}{dt}=m\cdot C_{H}\cdot e^{-\frac{qV_{s}}{kT}}\cdot {\left(-\frac{2\varepsilon n_{b}}{q}\cdot V_{s}\right)}^{\frac{1}{2}}$$

According to Equation (14), one can obtain:

(16) $$\frac{dN^{-}}{dV_{s}}=-\frac{1}{2}{\left(-\frac{2\varepsilon n_{b}}{qV_{s}}\right)}^{\frac{1}{2}}$$

Thus, the change of *N*^−^ with time becomes:

(17) $$\frac{dN^{-}}{dt}=\frac{dN^{-}}{dV_{s}}\cdot \frac{dV_{s}}{dt}=-\frac{1}{2}{\left(-\frac{2\varepsilon n_{b}}{qV_{s}}\right)}^{\frac{1}{2}}\cdot \frac{dV_{s}}{dt}$$

As Equations (11) and (17) are equal, with *u* = *qV*_s_/*kT*, we obtain

(18) $$u^{-1}\cdot e^{u}\cdot du=2m\cdot C_{H}\cdot dt$$

Define *u*_0_ and *u*_s_ to correspond to *qV*_s_/*kT* before H_2_ detection and *qV*_s0_/*kT* after detecting H_2_ gas for time *t*, respectively. Integrate both sides of Equation (18) in the time range of 0–*t* to obtain Equation (19).

(19) $$\int_{u_{0}}^{u_{s}}\frac{1}{u}\cdot e^{u}\cdot du=\int_{0}^{t}2m\cdot C_{H}\cdot dt$$

Let $E_{i}\left(x\right)=\int_{-\infty }^{x}e^{t}/tdt$, *a* = 2*m* and *b* = *E*i(*u*_0_), then Equation (19) and the expression of current become

(20) $$E_{i}\left(u_{s}\right)=a\cdot C_{H}\cdot t+b$$

(21) $$I=\beta \cdot e^{u_{s}}$$

**Figure 6:**
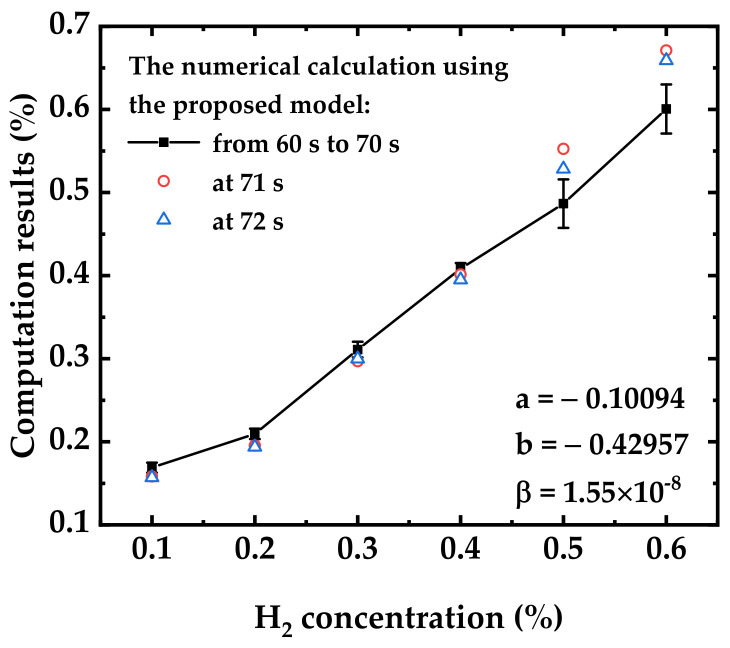
Fitting and calculation of hydrogen dynamic sensing under dry conditions. The black squares represent the fitting results obtained using the data in Figure 2a from 60 s to 70s. The red hollow circles and blue hollow triangles represent the concentration values calculated by substitute the current values from Figure 2a at 71 s and 72 s into the model (Equations (22) and (23)), respectively.

To simplify the calculation, the complex integral of *Ei*(*x*) was approximated as *e^x^*/*x*. Next, the current response data in Figure 2a from 60 s to 70 s were used for fitting to calculate *a*, *b*, and *β*, which can evade the errors caused by unstable experimental conditions, such as the changes of gases injected into the chamber at the beginning and the end of the response period. The final fitting results are shown in Figure 6, where *a* = −0.10094, *b* = −0.42957, and *β* = 1.55 × 10^−8^.

Thus, the final numerical model of hydrogen dynamic sensing under dry conditions is obtained as Equations (22) and (23).

(22) $$E_{i}\left(u_{s}\right)=-0.10094\cdot C_{H}\cdot t-0.42957$$

(23) $$I=1.55\times 10^{-8}\cdot e^{u_{s}}$$

To verify the model, the current values corresponding to the concentrations of each H_2_ sample at 71 s and 72 s in Figure 2a were substituted into the model. The calculated H_2_ concentrations were also plotted in Figure 6, as shown in the red hollow circles (71 s) and blue hollow triangles (72 s). The computation results using the data at 71 s and 72 s are in good agreement with the numerical model curve, except for a small error at 0.6% of H_2_.

#### 3.2.3. Analysis of Humidity Effects

When there are water molecules in the environment, they can also adsorb on the surface of In_2_O_3_, thus occupying the H adsorption site and reducing the concentration of the effective adsorption sites from *N^−^* to *N^−^*′ [42,43]. During the humid H_2_ sensing measurement in this paper, the humidity was kept at a certain RH level in each round of testing, so it can be assumed that the adsorption of water molecules on the surface of In_2_O_3_ was always in equilibrium in every humidity test. In addition to the detection of humid H_2_, the sensitivity to pure humidity of the Pt-In_2_O_3_ sensor was also tested (Figure S3 in the Supplementary Materials). There was no current response in the range of 0–15.5% RH, which indicates that there was no electron transfer between In_2_O_3_ and water molecules. Therefore, according to the Langmuir theory, the equilibrium coverage of water molecule is expressed as

(24) $$\theta =\frac{\alpha_{2}P}{1+P\;}$$

where *P* is the partial pressure of water vapor, and *α*_2_ is the Langmuir isotherm equilibrium constant. The relative humidity of the gas RH = *P*/*P*_s_, and *P*_s_ is the saturation vapor pressure, then, with *α*_2_ = *α*_2_·*P*_s_

(25) $$\theta =\frac{\alpha_{2}\cdot RH\cdot P_{s}}{1+\alpha_{2}\cdot RH\cdot P_{s}}=\frac{\alpha_{2}{}^{\prime }\cdot RH}{1+\alpha_{2}{}^{\prime }\cdot RH}$$

With defining $\Delta N_{max}^{-}$ as the total change of the adsorption site concentration for water molecules caused by 100% RH at room temperature, the change of adsorption site concentration $\Delta N^{-}$ at a certain RH level can be written as

(26) $$\Delta N^{-}=\Delta N_{max}^{-}\cdot \theta_{RH}=\Delta N_{max}^{-}\cdot \frac{\alpha_{2}{}^{\prime }\cdot RH}{1+\alpha_{2}{}^{\prime }\cdot RH}$$

Thus, the effective adsorption site concentration *N^−^*′ for H_2_ sensing is

(27) $$N^{-}{}^{\prime }=N^{-}-\Delta N^{-}=N^{-}-\Delta N_{max}^{-}\cdot \frac{\alpha_{2}{}^{\prime }\cdot RH}{1+\alpha_{2}{}^{\prime }\cdot RH}$$

Then, when the sensor started to detect humid H_2_ gas, by using the similar method in Section 3.2.2, according to Equations (9), (10), (14), (16) and (17), we can obtain

(28) $$\frac{dN^{-}}{dt}=-\alpha_{1}^{\prime }C_{H}N^{-}{}^{\prime }\cdot e^{-\frac{q_{0}-\Delta H+\left(E_{c}^{b}-E_{ss}\right)}{kT}}=m\cdot C_{H}\cdot \left(N^{-}-\Delta N^{-}\right)\cdot e^{-\frac{qV_{s}}{kT}}$$

(29) $$N^{-}{}^{\prime }={\left(-\frac{2\varepsilon n_{b}}{q}\cdot V_{s}\right)}^{\frac{1}{2}}-\frac{\alpha^{\prime }\cdot RH}{1+\alpha^{\prime }\cdot RH}\cdot \Delta N_{max}^{-}$$

(30) $$\frac{dN^{-}{}^{\prime }}{dV_{s}}=\frac{dN^{-}}{dV_{s}}=-\frac{1}{2}{\left(-\frac{2\varepsilon n_{b}}{qV_{s}}\right)}^{\frac{1}{2}}$$

(31) $$\frac{dV_{s}}{dt}=2m\cdot C_{H}\cdot e^{-\frac{qV_{s}}{kT}}\cdot \left[V_{s}+\Delta N^{-}\cdot {\left(-\frac{qV_{s}}{2\varepsilon n_{b}}\right)}^{\frac{1}{2}}\right]$$

It is found that |*dV*_s_/*dt*| decreases by comparing Equation (31) with the derivation in Section 3.2.2, indicating that the appearance of H_2_O slowed down the adsorption of H_2_. The above analysis on the effect of humidity is consistent with the test results in Figure 3.

## 4. Discussion

By using the dynamic sensing model obtain in Section 3.2.2 and Section 3.2.3, the behavior of the sensor can be predicted in real time, and the H_2_ concentration in the gas sample can be quickly calculated without waiting for the saturation of the sensor. For the errors in Figure 6, this section analyzed the main reasons and provided directions for further optimization of the sensing model as follows.

1. Concentrations and types of the reactive oxygen ions. During the hydrogen reduction reaction, the concentration of O^−^ adsorbed on the surface of In_2_O_3_ is continuously reduced, which leads to the strong chemical adsorption of additional $O_{x}^{\infty }$ and $O_{x}^{0}$ to generate $O_{x}^{-}$ and contributes to *dN^−^*/*dt*. In addition, it is assumed that the predominant ionic species reacting with H atom is O^−^ while modeling. However, $O_{2}^{-}$ may also participate in the sensing reactions, which can influence the indexes of the parameters in Equation (9).
2. Approximation of integral *Ei*. Integral *Ei* is one of the exponential integrals that gives solutions which cannot be evaluated using elementary functions. In order to obtain an analytical expression of dynamic current response, the function of *e^x^*/*x* was used to approximate *Ei*(*x*) in this article. The two functions are relatively close when |*x*| corresponding to |*qV*_s_/*kT*| is large, but as |*x*| gets closer to 0, the difference between the two becomes larger. Assuming that the initial value of *qV*_s_ in air is −1 eV, the relative error of *e^x^*/*x* and *Ei* is 2.57%. Whereas, if |*qV*_s_| is reduced by half after H_2_ sensing, the relative error of *e^x^*/*x* and *Ei* increases to 11.33%. Therefore, the more significant the reduction effect of hydrogen is (the higher H_2_ concentration), the more obvious the error of the proposed model becomes.
3. Experimental errors and noise effects. Firstly, the response of the sensor was weak for 0.1% H_2_, which was reflected in the deviation between the fitting calculation result and the real H_2_ centration of 0.1% in Figure 6. In addition, the oxygen in the gas samples also consume H_2_ under the catalysis of Pt, which may influence the response of the sensor and bring additional noise in the measurement results. Secondly, when the gas injected into the chamber is switched between the reference gas and the gas samples, it is inevitable that there are gas flow fluctuations at the beginning and end of the H_2_ sensing period, which can introduce unwanted fluctuations and errors into the test results. This is also the reason that only the relatively stable data in 60 s–70 s in Figure 2 were selected to perform data fitting in Section 3.2.2.
4. The effect of electric field. During the test, a fixed voltage was applied between the two electrodes of the device, thereby forming a certain electric field. This electric field makes the Fermi level of the sensitive material slightly different at different locations, which can also contribute to the inaccuracy of the model.

## 5. Conclusions

In summary, a Pt-In_2_O_3_ resistive sensor was investigated for H_2_ detection at room temperature. The sensor exhibits reliable sensitivities to variation of H_2_ concentration in the range from 0.1 to 0.6%. The current response of the sensor slightly decreased with the increasing humidity of the tested H_2_ samples because the water molecules partially occupied the sites for H_2_ reaction at the surface of In_2_O_3_. The sensing principle of the sensor was discussed in-depth, and a dynamic current sensing model was proposed. The H_2_ concentration of the tested gas sample can be figured out within relative short measurement time by using the dynamic model, which significantly reduces the sensing and recovery time of the sensor without waiting for the saturation of the response. Moreover, the model was also used to explain the effect of humidity on H_2_ sensitivity. Finally, the limitations of the proposed model and the reasons behind it were discussed. Future work could involve improving the dynamic sensing model from the aspects mentioned in the discussion, optimizing the inkjet-printed Pt-In_2_O_3_ sensor for better sensing properties, and establishing a robust sensing system on chip for real-time gas detection and identification.

## Figures and Tables

**Table 1:** Comparison of sensing performances towards hydrogen gas of sensors with various materials [6,26,27,28,29,30,31,32,33,34]. RT: room temperature, NW: nanowire, MOX: metal oxide, NF: nanofiber, R_a_: resistance in the air, R_g_: resistance in detection gas, –: not available. Adapted with permission from Ref. [27].

Sensor Materials	T (°C)	Detection Range	Response/Recovery Time (Response) at H_2_%	Advantage/Disadvantage	Ref.
Graphene				High surface-to-volume ratio and carrier mobility/difficult to control the content of oxygen functional groups [27]	
Pt/Graphene	200	10 ppm–0.1%	9/10 s (−) at 1%		[28]
Pt/ZnO/rGO	100	50–800 ppm	12/412 s (99%) at 400 ppm		[29]
Pd-metal				High sensitivity/humidity effect on the response and sensitivity, high electrical noise [27]	
Pd-Ni	RT	0.1–1%	7/30 s (2.75 kHz) at 0.1%		[30]
Pd/Co NWs	RT	0.1–3%	200/500 s (~0.2%) at 1%		[31]
MOX				High sensitivity and good stability/limited selectivity, humidity effect on the response and sensitivity [27]	
SnO_2_ NW	350	−	11/~17 s (4.5 V) at 400 ppm		[32]
ZnO NFs	350	0.1–10 ppm	400/~200 s (Ra/Rg = 74.7) at 100 ppb		[33]
Noble Metal-MOX				Low operating temperature, high sensitivity/humidity effect on the response and sensitivity [27]	
Pt/WO_3_	RT	0.2–1%	15 s/10 min (Ra/Rg = 18.5) at 1%		[6]
Pt–SnO_2_	350	100–500 ppm	29/36 s (Ra/Rg = ~60) at 100 ppm		[34]
Pt–In_2_O_3_ Nanoparticles	RT	0.1–0.6%	77/143 s (42.34%) at 0.6%		This work

## Data Availability

The datasets used and/or analyzed during the current study are available from the corresponding author on reasonable request.

## Supplementary Materials

The following supporting information can be downloaded at: https://www.mdpi.com/article/10.3390/s22197306/s1, Figure S1: SEM image and EDS analysis of the Pt-In_2_O_3_ sensing material; Figure S2: The response and recovery time of sensor based on the data in Figure 2b; Figure S3: Sensitivity to pure humidity of the inkjet-printed Pt-In_2_O_3_ sensor.

## References

[1] Abe J.O., Popoola A.P.I., Ajenifuja E., Popoola O.M. (2019). Hydrogen energy, economy and storage: Review and recommendation. Int. J. Hydrogen Energy.

[2] Lu S., Zhang Y., Liu J., Li H.-Y., Hu Z., Luo X., Gao N., Zhang B., Jiang J., Zhong A. (2021). Sensitive H_2_ gas sensors based on SnO_2_ nanowires. Sens. Actuators B.

[3] Mao X., Ying R., Yuan Y., Li F., Shen B. (2021). Simulation and analysis of hydrogen leakage and explosion behaviors in various compartments on a hydrogen fuel cell ship. Int. J. Hydrogen Energy.

[4] Zhang X.-Y., Ma R.-H., Li L.-S., Fan L., Yang Y.-T., Zhang S.-Y. (2021). A room-temperature ultrasonic hydrogen sensor based on a sensitive layer of reduced graphene oxide. Sci. Rep..

[5] Wang F., Hu K., Liu H., Zhao Q., Wang K., Zhang Y. (2020). Low temperature and fast response hydrogen gas sensor with Pd coated SnO_2_ nanofiber rods. Int. J. Hydrogen Energy.

[6] Lee J., Koo H., Kim S.Y., Kim S.J., Lee W. (2021). Electrostatic spray deposition of chemochromic WO_3_-Pd sensor for hydrogen leakage detection at room temperature. Sens. Actuators B.

[7] Lee J.-H., Kim J.-H., Kim J.-Y., Mirzaei A., Kim H.W., Kim S.S. (2019). ppb-Level Selective Hydrogen Gas Detection of Pd-Functionalized In_2_O_3_-Loaded ZnO Nanofiber Gas Sensors. Sensors.

[8] Li H., Wu C.-H., Liu Y.-C., Yuan S.-H., Chiang Z.-X., Zhang S., Wu R.-J. (2021). Mesoporous WO_3_-TiO_2_ heterojunction for a hydrogen gas sensor. Sens. Actuators B.

[9] Le H.-J., van Dao D., Yu Y.-T. (2020). Superfast and efficient hydrogen gas sensor using PdAu alloy @ZnO core–shell nanoparticles. J. Mater. Chem. A.

[10] Ambardekar V., Bhowmick T., Bandyopadhyay P.P. (2022). Understanding on the hydrogen detection of plasma sprayed tin oxide/tungsten oxide (SnO_2_/WO_3_) sensor. Int. J. Hydrogen Energy.

[11] Inyawilert K., Wisitsoraat A., Liewhiran C., Tuantranont A., Phanichphant S. (2019). H_2_ gas sensor based on PdO_x_-doped In_2_O_3_ nanoparticles synthesized by flame spray pyrolysis. Appl. Surf. Sci..

[12] Zheng Z.Q., Zhu L.F., Wang B. (2015). In_2_O_3_ nanotower hydrogen gas sensors based on both schottky junction and thermoelectronic emission. Nanoscale Res. Lett..

[13] Hu J., Sun Y., Xue Y., Zhang M., Li P., Lian K., Zhuiykov S., Zhang W., Chen Y. (2018). Highly sensitive and ultra-fast gas sensor based on CeO_2_-loaded In_2_O_3_ hollow spheres for ppb-level hydrogen detection. Sens. Actuators B.

[14] Tonezzer M. (2019). Selective gas sensor based on one single SnO_2_ nanowire. Sens. Actuators B.

[15] Song L., Yang L., Wang Z., Liu D., Luo L., Zhu X., Xi Y., Yang Z., Han N., Wang F. (2019). One-step electrospun SnO_2_/MO_x_ heterostructured nanomaterials for highly selective gas sensor array integration. Sens. Actuators B.

[16] Umar A., Ammar H.Y., Kumar R., Almas T., Ibrahim A.A., AlAssiri M.S., Abaker M., Baskoutas S. (2020). Efficient H_2_ gas sensor based on 2D SnO_2_ disks: Experimental and theoretical studies. Int. J. Hydrogen Energy.

[17] Deng L., Bao L., Xu J., Wang D., Wang X. (2020). Highly sensitive acetone gas sensor based on ultra-low content bimetallic PtCu modified WO_3_·H_2_O hollow sphere. Chin. Chem. Lett..

[18] Mohl M., Dombovari A., Szabó M., Järvinen T., Pitkänen O., Sápi A., Juhász K.L., Kéri A., Galbács G., Kukovecz Á. (2019). Size-dependent H_2_ sensing over supported Pt nanoparticles. J. Nanosci. Nanotechnol..

[19] Thai N.X., van Duy N., van Toan N., Hung C.M., van Hieu N., Hoa N.D. (2020). Effective monitoring and classification of hydrogen and ammonia gases with a bilayer Pt/SnO_2_ thin film sensor. Int. J. Hydrogen Energy.

[20] Chen Z., Hu K., Yang P., Fu X., Wang Z., Yang S., Xiong J., Zhang X., Hu Y., Gu H. (2019). Hydrogen sensors based on Pt-decorated SnO_2_ nanorods with fast and sensitive room-temperature sensing performance. J. Alloys Compd..

[21] Wang D., Yang J., Bao L., Cheng Y., Tian L., Ma Q., Xu J., Li H.-J., Wang X. (2021). Pd nanocrystal sensitization two-dimension porous TiO_2_ for instantaneous and high efficient H_2_ detection. J. Colloid Interface Sci..

[22] Wang Y., Liu B., Cai D., Li H., Liu Y., Wang D., Wang L., Li Q., Wang T. (2014). Room-temperature hydrogen sensor based on grain-boundary controlled Pt decorated In_2_O_3_ nanocubes. Sens. Actuators B.

[23] Neri G., Bonavita A., Micali G., Rizzo G., Galvagno S., Niederberger M., Pinna N. (2005). A highly sensitive oxygen sensor operating at room temperature based on platinum-doped In_2_O_3_ nanocrystals. Chem. Commun..

[24] Hung C.M., Vuong V.A., van Duy N., van An D., van Hieu N., Kashif M., Hoa N.D. (2020). Controlled growth of vertically oriented trilayer MoS_2_ nanoflakes for room-temperature NO_2_ gas sensor applications. Phys. Status Solidi A.

[25] Zhang X., Sun J., Tang K., Wang H., Chen T., Jiang K., Zhou T., Quan H., Guo R. (2022). Ultralow detection limit and ultrafast response/recovery of the H_2_ gas sensor based on Pd-doped rGO/ZnO-SnO_2_ from hydrothermal synthesis. Microsyst. Nanoeng..

[26] Shi Y., Xu H., Liu T., Zeb S., Nie Y., Zhao Y., Qin C., Jiang X. (2021). Advanced development of metal oxide nanomaterials for H_2_ gas sensing applications. Mater. Adv..

[27] Wang B., Sun L., Schneider-Ramelow M., Lang K.-D., Ngo H.-D. (2021). Recent advances and challenges of nanomaterials-based hydrogen sensors. Micromachines.

[28] Phan D.-T., Youn J.-S., Jeon K.-J. (2019). High-sensitivity and fast-response hydrogen sensor for safety application using Pt nanoparticle-decorated 3D graphene. Renew. Energy.

[29] Drmosh Q.A., Yamani Z.H., Hendi A.H., Gondal M.A., Moqbel R.A., Saleh T.A., Khan M.Y. (2019). A novel approach to fabricating a ternary rGO/ZnO/Pt system for high-performance hydrogen sensor at low operating temperatures. Appl. Surf. Sci..

[30] Wang W., Liu X., Mei S., Liu M., Lu C., Lu M. (2019). Development of a high stability Pd-Ni alloy thin-film coated saw device for sensing hydrogen. Sensors.

[31] Du L., Feng D., Xing X., Fu Y., Fonseca L.F., Yang D. (2019). Palladium/cobalt nanowires with improved hydrogen sensing stability at ultra-low temperatures. Nanoscale.

[32] Zhu L., Zeng W., Li Y. (2019). A non-oxygen adsorption mechanism for hydrogen detection of nanostructured SnO_2_ based sensors. Mater. Res. Bull..

[33] Kim J.-H., Mirzaei A., Woo Kim H., Kim S.S. (2019). Combination of Pd loading and electron beam irradiation for superior hydrogen sensing of electrospun ZnO nanofibers. Sens. Actuators B.

[34] Yin X.-T., Zhou W.-D., Li J., Wang Q., Wu F.-Y., Dastan D., Wang D., Garmestani H., Wang X.-M., Ţălu Ş. (2019). A highly sensitivity and selectivity Pt-SnO_2_ nanoparticles for sensing applications at extremely low level hydrogen gas detection. J. Alloys Compd..

[35] Ou Y., Zhu G., Liu P., Jia Y., Zhu L., Nie J., Zhang S., Zhang W., Gao J., Lu H. (2022). Anchoring Platinum Clusters onto Oxygen Vacancy-Modified In_2_O_3_ for Ultraefficient, Low-Temperature, Highly Sensitive, and Stable Detection of Formaldehyde. ACS Sens..

[36] Hwang J., Jung H., Shin H.-S., Kim D.-S., Kim D.S., Ju B.-K., Chun M. (2021). The Effect of Noble Metals on Co Gas Sensing Properties of In_2_O_3_ Nanoparticles. Appl. Sci..

[37] Marikutsa A., Rumyantseva M., Konstantinova E.A., Gaskov A. (2021). The Key Role of Active Sites in the Development of Selective Metal Oxide Sensor Materials. Sensors.

[38] Barsan N., Simion C., Heine T., Pokhrel S., Weimar U. (2010). Modeling of sensing and transduction for p-type semiconducting metal oxide based gas sensors. J. Electroceram..

[39] Barsan N., Weimar U. (2001). Conduction model of metal oxide gas sensors. J. Electroceram..

[40] Suematsu K., Sasaki M., Ma N., Yuasa M., Shimanoe K. (2016). Antimony-doped tin dioxide gas sensors exhibiting high stability in the sensitivity to humidity changes. ACS Sens..

[41] Bejaoui A., Guerin J., Aguir K. (2013). Modeling of a p-type resistive gas sensor in the presence of a reducing gas. Sens. Actuators B.

[42] Rasch F., Postica V., Schütt F., Mishra Y.K., Nia A.S., Lohe M.R., Feng X., Adelung R., Lupan O. (2020). Highly selective and ultra-low power consumption metal oxide based hydrogen gas sensor employing graphene oxide as molecular sieve. Sens. Actuators B.

[43] Sun M., Yu H., Dong X.-T., Xia L., Yang Y. (2020). Sedum lineare flower-like ordered mesoporous In_2_O_3_/ZnO gas sensing materials with high sensitive response to H_2_S at room temperature prepared by self-assembled of 2D nanosheets. J. Alloys Compd..

