# Supplementary material for: Characterization and Modeling of a Pt-In2O3 Resistive Sensor for Hydrogen Detection at Room Temperature
Source: Sensors (Basel). 2022 Sep 26;22(19):7306. doi: 10.3390/s22197306 (PMC9573015; doi:10.3390/s22197306)
Supplement: Supplementary file 1 [file sensors-22-07306-s001.zip › sensors-1925388-supplementary.pdf]

Supplementary information for

Characterization and Modeling of a Pt-In_2O_3 Resistive Sensor for Hydrogen Detection at Room Temperature

Meile Wu *, Zebin Wang, Zhanyu Wu, Peng Zhang, Shixin Hu, Xiaoshi Jin, Meng Li and Jong-Ho Lee

Correspondence: meilwu@sut.edu.cn

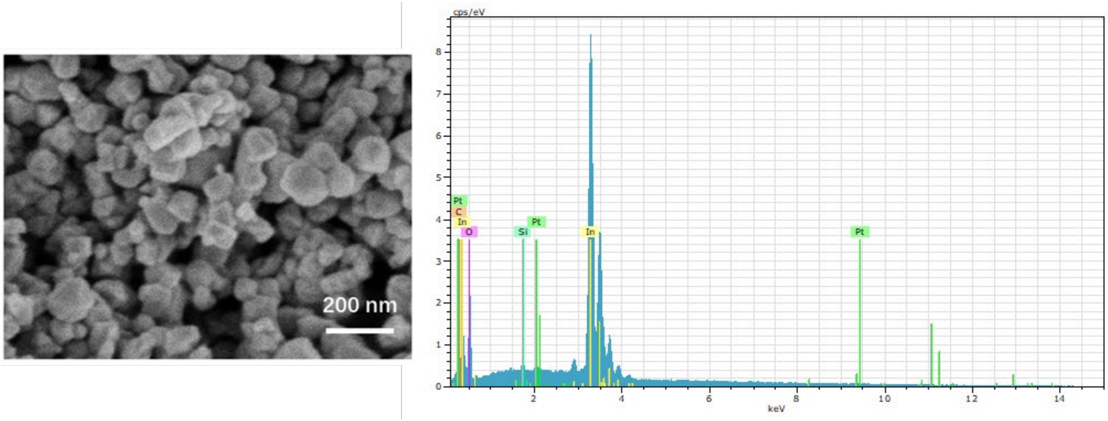

(**a**) (**b**)

Figure S1. SEM image and EDS analysis of the Pt-In_2O_3 sensing material.

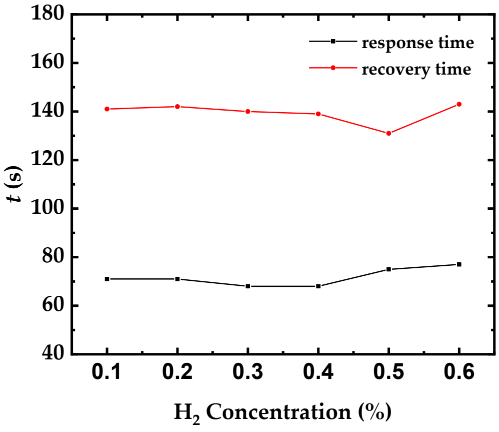

Figure S2. The response and recovery time of sensor based on the data in Figure 2b. The response time and recovery time of the sensor were defined as the time required for the current to change to 90% of its final value.

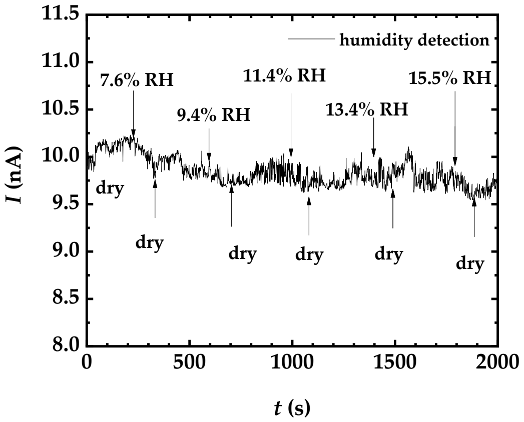

Figure S3. Sensitivity to pure humidity of the inkjet-printed Pt-In_2O_3 sensor.
